# Comparison of thulium laser *en bloc* resection of bladder tumor and transurethral resection of bladder tumor on catheter-related bladder discomfort- a single center experience

**DOI:** 10.3389/or.2025.1653825

**Published:** 2025-11-12

**Authors:** Wenbo Gao, Haihon Ye, Jiawen Huang, Telei Chen

**Affiliations:** Department of Urology, Ningbo Urology and Nephrology Hospital, Ningbo, Zhejiang, China

**Keywords:** bladder cancer, thulium laser, *en bloc* resection, catheter-related bladderdiscomfort, transurethal resection of bladder tumor

## Abstract

**Objective:**

To Compare the effects between thulium laser *en bloc* resection of bladder tumor (ERBT) and conventional transurethral resection of bladder tumor (TURBT) on catheter-related bladder discomfort (CRBD) in patients with bladder cancer.

**Methods:**

Between January 2022 and December 2024, we retrospectively collected the demographic and clinical data for patients with bladder cancer. A total of 79 patients in the conventional TURBT group and 58 patients in the thulium laser ERBT group completed the study. Both demographic and outcome variables were recorded; and we compared the incidence and severity of CRBD at 1, 6 and 24 h postoperatively, score of postoperative pain at 1, 6 and 24 h and patient satisfaction at 24 h following the surgery.

**Results:**

There were no significant differences in age, gender proportion, tumor multiplicity, tumor size and location, and duration of surgery between the two groups (*P >* 0.05). Pathological examination revealed that the ERBT had a higher rate of detrusor presence than TURBT (*P* = 0.04). The incidence and severity of postoperative CRBD were lower in ERBT group than TURBT group at 1 and 6h (*P* < 0.001), while there were no statistically significant difference between the two groups (*P* = 0.17) at 24 h. The VAS scores of postoperative pain were significantly lower in ERBT group than in TURBT group at 1 and 6 h postoperatively (*P* = 0.001 and *P* = 0.02, respectively). But at 24 h, there was no statistically significant difference (*P* = 0.08). As to postoperative patient satisfaction at 24 h, the result of ERBT group was significantly lower than TURBT group (*P* = 0.02). Additionally, the ERBT group had significantly less intraoperative blood loss and shorter postoperative irrigation duration (*P* = 0.001). No significant difference was found in the duration of indwelling catheter between the two groups (*P* = 0.07).

**Conclusion:**

The results suggest that compared to conventional TURBT, thulium laser ERBT significantly reduce CRBD incidence and severity, lower postoperative pain, and improve postoperative patient satisfaction. However, as a single-center retrospective study, these findings require further validation by large-scale, prospective, multicenter trials.

## 1 Introduction

The urethral catheters are used widely in clinical practice, with an estimated 15%–25% of hospitalized patients undergoing indwelling catheter placement (1). In particular, about 80% of urological patients have used urethral catheters, which brought great convenience to the diagnosis and treatment. However, transurethral catheterization introduces mechanical irritation to the bladder mucosa and urethral epithelium. This iatrogenic stimulus-usually referred as catheter-related bladder discomfort (CRBD)- is a distressing and undesirable complication that may result in disastrous consequences, such as urinary frequency, burning sensations, constant urge to catheter manipulation. Or even interfering with surgical outcomes: severe bladder spasms can dislodge hemostatic clots, resulting in secondary hemorrhage, delayed wound healing and influencing the patient’s recovery process. Clinical studies revealed that the incidence of CRBD varied between 47% and 93% following transurethral procedures (2).

Although sometimes similar to that of overactive bladder (OAB) in symptoms, CRBD is unique in the etiology. Its pathophysiology is now considered as mechanical, inflammatory (3) and chemical irritation of bladder mucosa by indwelling catheters, activating M3 muscarinic receptors and triggering involuntary detrusor contractions. These symptoms not only compromise patient satisfaction but also increase analgesic consumption and other adverse outcomes. Thus, the treatment of CRBD poses a particularly significant challenge in post-transurethral resection patients, when catheterization is crucial for postoperative irrigation and avoidance of bleeding. Multimodal prophylactic strategies reported in the literature include lubricants containing local anesthetic such as lidocaine or tetracaine being instilled into the urethra or applied on the catheter (4), catheter optimization (reducing the balloon volume or different catheter fixation sites) (5), transcutaneous electrical nerve stimulation (TENS) and acupuncture (6), as well as administration of drugs with different mechanisms, for instance, histamine H1-receptor antagonist (7), nonsteroidal anti-inflammatory drugs (NSAIDs) (8), cyclooxygenase-2 selective NSAIDs, tolterodine, tramadol (9), etc. Many studies have shown their abilities to prevent CRBD to certain extent; but the real-world clinical application results were not fully satisfactory, or side effects usually occur, such as dry mouth, sedation, nausea, or vomiting (10). No efficient management for CRBD without undesirable consequences has been established yet (11).

As the gold standard for non-muscle invasive bladder cancer (NMIBC) management, conventional transurethral resection of bladder tumor (TURBT) employs high-frequency electrocautery to resect tumors in a piecemeal fashion. The principal aims of TURBT include establishment of the histologic diagnosis, offering therapeutic protocols and guiding prognosis, among which the accurate pathological diagnosis is fundamental. However, multiple negative factors, including the inherent limitations-extensive thermal injury to adjacent tissues, irregular resection of the bladder wall and margins, and prolonged intraoperative bleeding, together with continued irritation of the bladder wall by reflux fluid (5), aggravate postoperative bladder mucosal inflammation. Such inflammation potentiates CRBD by sensitizing afferent nerve fibers and upregulating nociceptive signaling pathways. While pharmacological interventions (e.g., anticholinergics, lidocaine gel) provide symptomatic relief (12), they fail to address the root cause: surgical trauma. In addition, well-recognized risks might be encountered during TURBT, such as destruction of the tumor integrity, obturator nerve reflex, difficult control of cutting depth and bladder perforation (13). Thus, researchers are seeking better methods to make up for these drawbacks, trying to improve treatment strategies and reduce the risks.

Thulium laser *en bloc* resection of bladder tumor (ERBT) has emerged as a promising alternative. Operating at a wavelength of 1.75–2.22 μm (average 1.940 μm), its tissue penetration depth is 250 μm and thermal damage depth is only 200 μm. The thulium laser transurethral resection achieves rapid and precise tissue resection and vaporization with minimal collateral thermal injury. Moreover, it has excellent hemostasis performance which makes a thin and plain cutting surface (14). Both theoretical and clinical studies showed that ERBT technique removes bladder tumor in its entirety, encompassing the tumor and base (“a no-touch technique”), with deep enough resection for the underneath detrusor muscle (DM). So this technique better complies with the oncological criteria of “optimized resection with low residual tumor rates” for cancer treatment (15). ERBT not only preserves tumor architecture for accurate pathological staging (16), but also eliminates repeated surgical instrument passing into the bladder, thereby minimizing mechanical irritation.

Yet, from the current data, it remains unclear as to the impact of thulium laser ERBT on CRBD. In the present study, we compared the effects of thulium laser ERBT and conventional TURBT on the incidence and severity of CRBD, and further evaluated postoperative pain and patient satisfaction, with the aim to clarify whether thulium laser ERBT had superiority in alleviating postoperative CRBD over conventional TURBT.

## 2 Materials and methods

We retrospectively collected the demographic and clinical data for patients undergoing thulium laser ERBT and conventional TURBT between January 2022 and December 2024 in our hospital.

The eligibility criteria included patients with an age range of 40–82 years, primary Ta or T1 NMIBC on pathology. Exclusion criteria were a history of bladder disease (such as neurogenic bladder, bladder outflow obstruction, or hyperactive bladder), with concurrent upper tract urothelial tumor, pure carcinoma *in situ* or suspected invasion into muscle as seen on computed tomography (CT), magnetic resonance imaging (MRI) or pathology, or recurrent NMIBC. The study protocol was approved by the Institutional Review Board of our hospital, and informed written consent was obtained from all patients. The conventional TURBT was performed using bipolar electrocautery in a piecemeal fashion, which is the standard technique at our hospital. The thulium laser ERBT was performed with an *en bloc* procedure as described below.

### 2.1 Surgical technique of thulium laser ERBT

The surgical procedures were performed by two experienced senior surgeons who adhered to internally standardized procedures.

During the operation, the patient was in the lithotomy position under general or continuous epidural anesthesia, with a 26 FR resectoscope and continuous normal saline irrigation. The power of the thulium laser was set at 25–30W, which was adjusted during the operation according to the volume of the tumor and the site of the surgical resection.

**FIGURE 1:**
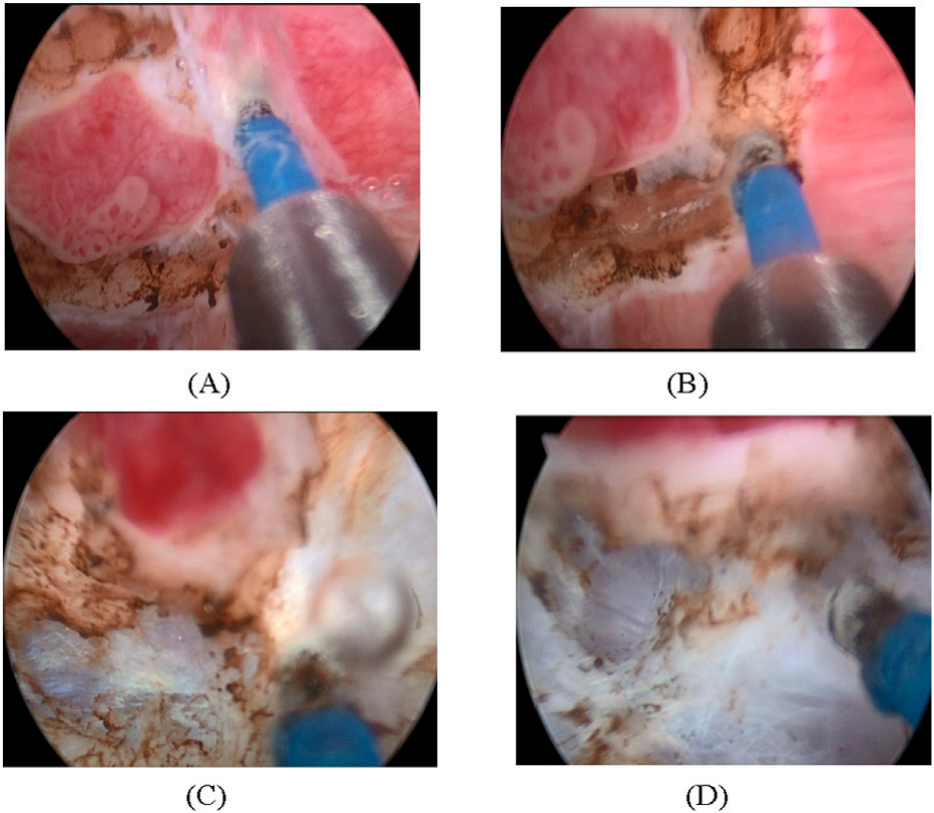
Procedures of thulium laser ERBT. **(A)** circumferential incision 5–10 mm away from the tumor base. **(B)** resection proceeding to the deep muscle layer. **(C)** demonstration of deep muscle layer. **(D)** *en bloc* resection almost finished.

The surgical steps of thulium laser ERBT have been described in detail in our previous articles (15, 16). Briefly, a laser fiber was introduced in the working sheath of a continuous-flow resectoscope. After careful observation of the whole bladder and tumors, a circumferential incision was performed around the tumor with a safety margin of approximately 5 mm. The resection proceeded toward the deeper muscle layers, combining laser incision with blunt dissection of the resectoscope tip, then progressive detachment of the lesion followed by exposing and lifting the tumor base. This “traction-and-countertraction” technique facilitates exposure of the surgical plane and helps prevent bladder perforation. At the visible anatomical level, the muscle fibers were cut from the base of the tumor in different directions. In case of minor bleeding, coagulation was achieved with laser. Gradually the entire tumor was removed with detrusor muscle beneath the base. The whole tumor specimen, along with its base, was retrieved entirely through the resectoscope sheath, which was the final and important step. When the tumor size was >3 cm, it was necessary to incise longitudinally into two or more parts. During the surgery, it is important that the deep muscle layer is identified, so as to avoid perforation or losing vision. The surgical procedures are illustrated in Figure 1.

Upon completion of the surgery, a 20 FR sterile Foley catheter was inserted, and 15 mL of distilled water was injected into the balloon. The patient was returned to the ward after recovery from anesthesia. Postoperative bladder irrigation ceased immediately after gross hematuria disappeared. Tumor specimens were sent for histopathological examination. Two groups of patients received bladder instillation with Mitomycin-C at 4–6 days postoperatively at a dose of 40mg/50 mL of saline.

### 2.2 Outcome measures

All patients were given elaborate instructions regarding CRBD and the pain scoring method to be used post-operatively by a dedicated research nurse who was blinded to the surgical assignment. The primary outcome of the study was the incidence and severity of CRBD at 1, 6 and 24 h after transurethral surgery. CRBD was defined as a burning sensation at urethra with an urge to void, urinary frequency and painful discomfort in the supra-pubic region. The severity of CRBD symptoms was recorded form mild to severe using a scale that has been widely used and validated in previous studies on post-TURBT CRBD (12): ① mild-discomfort reported only when asked; ② moderate-complaint of discomfort independent of questioning and not accompanied by behavioral response; ③ severe-discomfort reported by patients on their own together with behavioral responses, such as intense vocal response, flailing limbs, and attempts to remove the catheter (17).

The secondary outcomes of the study included score of postoperative pain, which was defined as tingling discomfort at the suprapubic region, at 1, 6 and 24 h postoperatively; as well as patient satisfaction at 24 h following the surgery. The evaluation of pain was achieved by use of the Visual Analog Scale (VAS) score-a well-validated and reliable tool for measuring acute pain (12, 17), which included a straight line labeling 0–10 cm in length. A score of 0 = no pain, 3 = normal sleep and tolerable pain, 5 = inability to fall asleep and intolerable pain, 8 = passive position and autonomic dysfunction, and 10 = the worst and unendurable pain. The patients were instructed to mark a point on the line that best expressed their pain (11). The definitions were carefully explained to help patients distinguish between the suprapubic surgical pain (a constant, dull ache) and CRBD (a burning sensation with urge to void and discomfort perceived in the urethra or supra-pubic region). Patient satisfaction was evaluated with a four-point Likert scale ranging from 1 = very satisfied, 2 = somewhat satisfied, 3 = somewhat dissatisfied to 4 = very dissatisfied (8).

**FIGURE 2:**
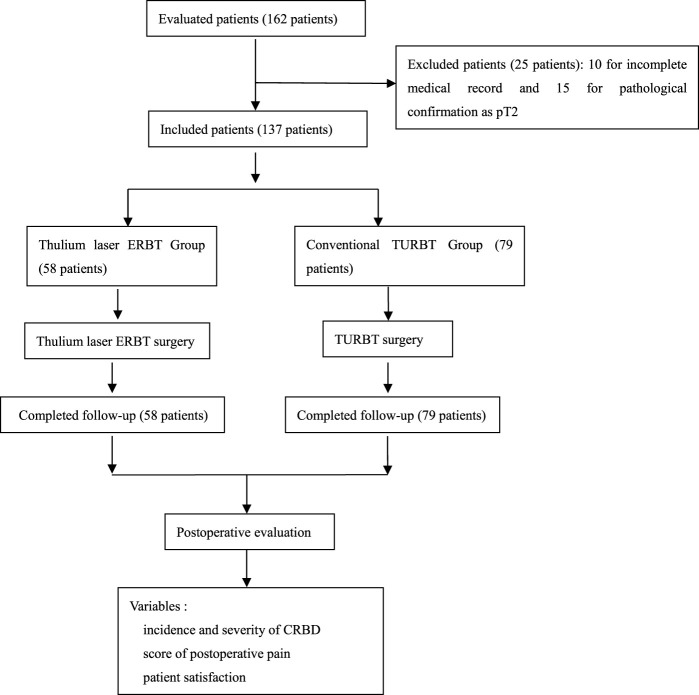
Study flow chart.

Both demographic and outcome variables were recorded. All methods were conducted in accordance with relevant guidelines and regulations including the Declaration of Helsinki. The study was approved by the Institutional Ethics Committee of our hospital. All patients have read and signed the informed consent. The study flow chart is seen in Figure 2. Since the main purpose of this study was to compare CRBD between thulium laser ERBT and conventional TURBT in patients with NMIBC, long-term oncological outcomes and patient survival were not reported here.

### 2.3 Statistical analysis

The SPSS version 18.0 (SPSS Inc., USA) was used to conduct statistical analysis. The results are expressed as number (%) or mean ± standard deviation. Variables with normal distribution were compared for intergroup differences using Student’s t*-*test. Categorical variables were compared for intergroup differences using Chi-square test. A *P* value <0.05 was considered statistically significant for all analyses.

## 3 Results

In this study, a total of 162 patients were enrolled, among which 25 were excluded due to incomplete medical record, or final pathological confirmation as pT2 (as depicted in Figure 2). Eventually, 79 patients in the conventional TURBT group and 58 patients in the thulium laser ERBT group completed the study.

**FIGURE 3:**
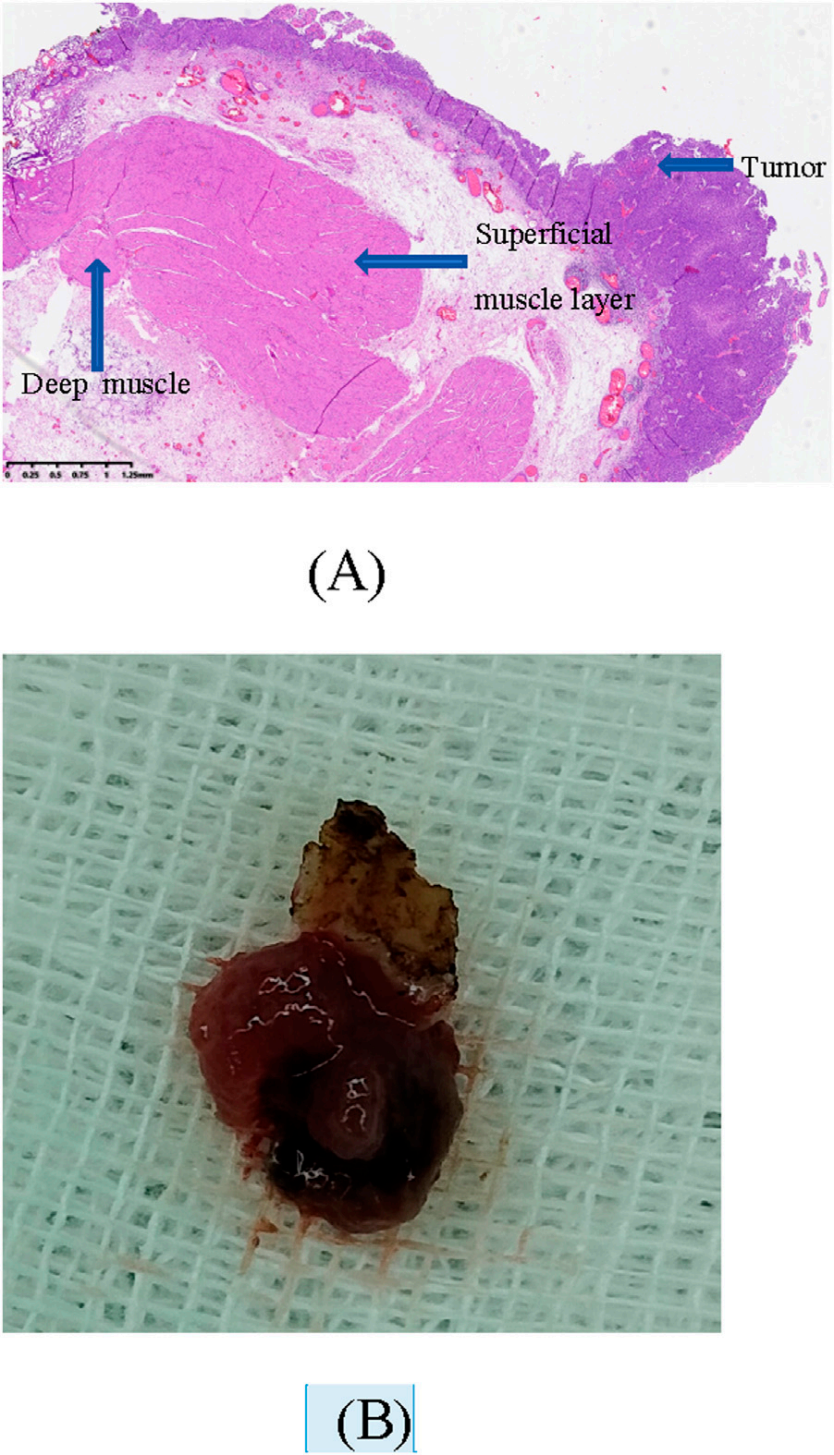
A histopathological image of thulium laser ERBT specimen and a photo of postoperative gross specimen. **(A)** H&E staining image of a specimen (2 × 10). **(B)** Postoperative gross specimen.

The baseline characteristics and intraoperative variables of the patients in both groups were seen in Table 1. As shown in the table, the age, gender proportion, tumor multiplicity, tumor size and location were similar between the two groups (*P >* 0.05). The duration of surgery in ERBT group was longer than TURBT group, although there was no statistically significant difference between the two groups (30.9 ± 8.8 vs. 28.2 ± 8.0 min, *P* = 0.06). No intraoperative complications occurred with thulium laser ERBT (except one case of bladder perforation which was performed at the initial stage of this surgery). But for conventional TURBT, there were 7 cases of obturator nerve reflex and 8 cases of bladder perforation. The ERBT group had significantly less intraoperative blood loss (25.3 ± 8.1 mL and 48.6 ± 12.4 mL, respectively, *P* = 0.001). Pathological examination revealed that the ERBT had a higher rate of detrusor presence than TURBT (91.4% vs. 78.5%, *P* = 0.04). A histopathological image of thulium laser ERBT specimen and a photo of postoperative gross specimen are shown in Figure 3. With regard to the postoperative irrigation duration, the results were 9.9 ± 1.9 h in ERBT group and 11.1 ± 2.5 h in TURBT group, respectively, with a significant difference between the two groups (*P* = 0.001).

**TABLE 1:** Baseline characteristics and intraoperative variables of the study population.

Variable	ERBT group	TURBT group	*P* value
	(*n* = 58)	(*n* = 79)	
Age (years)	67.5 ± 8.5	65.8 ± 8.1	0.26
Male/female	47/11	55/24	0.13
Duration of surgery (minutes)	30.9 ± 8.8	28.2 ± 8.0	0.06
Tumor size, number (%)			
<3 cm	44 (75.9%)	64 (81.0%)	0.46
>3 cm	14 (24.1%)	15 (19.0%)	
Solitary/multiple mass	50/8	69/10	0.85
Tumor location, number (%)			0.56
Lateral wall	33 (48.5%)	39 (42.4%)	
Anterior wall	6 (8.8%)	9 (9.8%)	
Posterior wall	11 (16.2%)	15 (16.3%)	
Bladder neck	7 (10.3%)	12 (13.0%)	
Trigone	8 (11.8%)	10 (10.9%)	
Dome	3 (4.4%)	7 (7.6%)	
Obturator nerve reflex (%)	0	7 (8.9%)	0.02
Bleeding volume during operation (mL)	25.3 ± 8.1	48.6 ± 12.4	0.001
Bladder perforation (%)	1 (1.7%)	8 (10.1%)	0.04
Detrusor presence (%)	53 (91.4%)	62 (78.5%)	0.04
Postoperative irrigation duration (hours)	9.9 ± 1.9	11.1 ± 2.5	0.001
Duration of indwelling urinary catheter (days)	2.1 ± 0.5	2.4 ± 0.6	0.07

The incidence of postoperative CRBD (mild, moderate, or severe) was lower in ERBT group as compared with TURBT group at 1 and 6h (Table 2, *P* < 0.001). The severity of CRBD was also decreased in ERBT group than in TURBT group at 1 and 6h (*P* < 0.001). However, at 24h, the incidence and severity of CRBD were not statistically significant different between the two groups (*P* = 0.17).

**TABLE 2:** The incidence and severity of postoperative CRBD in the study population.

CRBD	ERBT group (*n* = 58)	TURBT group (*n* = 79)	*P* value
Postoperative hours			
1 h			
Incidence (%)	19 (32.8%)	67 (84.8%)	<0.001
Severity			
Mild	11 (19.0%)	14 (17.7%)	
Moderate	6 (10.3%)	38 (48.1%)	
Severe	2 (3.4%)	15 (19.0%)	
6 h			
Incidence	12 (20.7%)	60 (75.9%)	<0.001
Severity			
Mild	8 (13.8%)	18 (22.8%)	
Moderate	3 (5.2%)	32 (40.5%)	
Severe	1 (1.7%)	10 (12.6%)	
24 h			
Incidence	9 (15.5%)	20 (25.3%)	0.17
Severity			
Mild	7 (12.1%)	13 (16.4%)	
Moderate	2 (3.4%)	6 (7.6%)	
Severe	0	1 (1.3%)	

**FIGURE 4:**
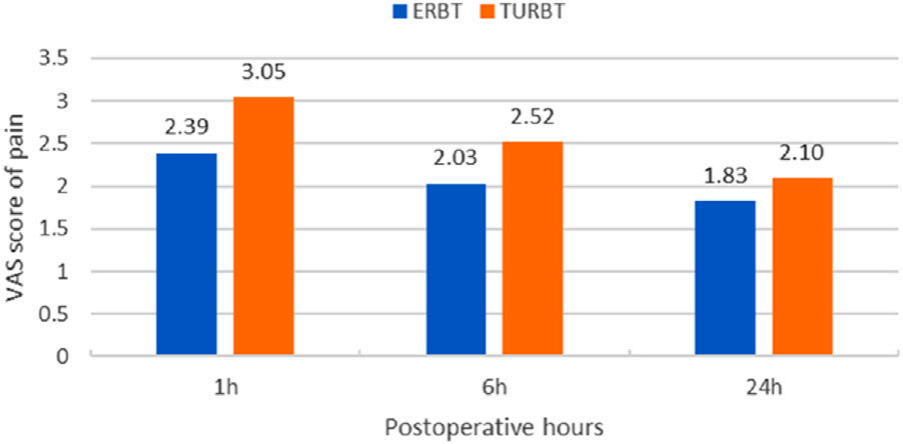
The VAS scores of postoperative pain in the study population.

The VAS scores of postoperative pain were significantly lower in ERBT group than in TURBT group at 1 and 6h postoperatively (1h: 2.39 ± 1.02 vs. 3.05 ± 1.25, *P* = 0.001; 6h: 2.03 ± 1.05 vs. 2.52 ± 1.26, *P* = 0.02, Figure 4). But at 24h, there was no statistically significant difference between the two groups (1.83 ± 0.96 vs. 2.10 ± 0.83, *P* = 0.08).

**FIGURE 5:**
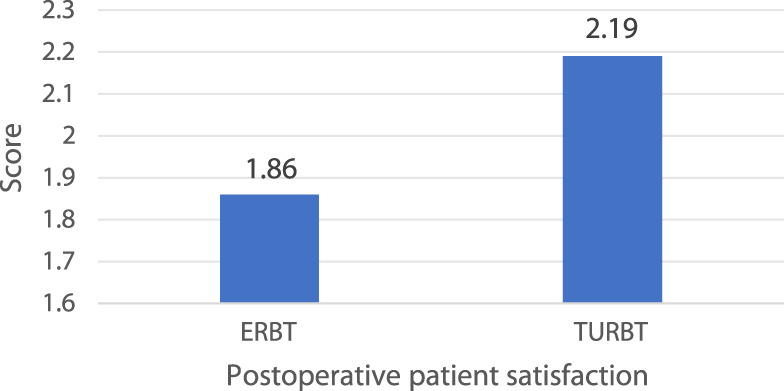
The postoperative patient satisfaction scores in the study population.

Concerning postoperative patient satisfaction at 24h which was evaluated with a four-point Likert scale, the result of ERBT group was significantly lower than that of the TURBT group (1.86 ± 0.73 vs. 2.19 ± 0.83, *P* = 0.02; Figure 5), suggesting that the thulium laser ERBT patients were more satisfied with their surgery.

## 4 Discussion

Postoperative CRBD causes significant distress for patients with urinary catheter and is often accompanied by behavioral reactions, such as agitation, intense verbal reaction, flailing limbs, and even attempts to pull out the catheter, potentially leading to urethral injury and urethral strictures. CRBD not only worsens postoperative pain and anxiety, triggering postoperative cognitive dysfunction and reducing patient satisfaction, but also increases healthcare staff workload, as it might prolong hospital stays and place unnecessary strain on healthcare resources.

The precise mechanisms underlying CRBD after TURBT remain incompletely understood. Current evidence indicates that multiple reasons might play role in the CRBD symptoms: ① Compression by the Foley catheter balloon on the bladder neck and trigone. This stimulates nerve endings, generating electrical impulses that travel via the lumbar nerves and spinal cord. These impulses then reach the central nerve sensory areas, rendering pain and various types of discomfort. ② Over-inflation of the catheter balloon, increasing pressure on the bladder neck and internal urethral orifice (11). This readily triggers bladder spasms, causing the patient pain, discomfort, and an urgent sensation to urinate. ③ Low pain threshold in some patients, making them unable to tolerate the local irritation of the urethra caused by the indwelling catheter. ④ Lack of understanding about the importance of the indwelling catheter and its management in patients, which aggravates the patients’ discomfort and psychological distress. And importantly ⑤ trauma-induced inflammation: catheter insertion causes mechanical injury to the bladder/urethral mucosa, especially after piecemeal resection of the tumor (17). Damaged urothelium releases pro-inflammatory mediators and damage-associated molecular patterns (DAMPs), triggering innate immune responses (e.g., neutrophil/macrophage recruitment) and release of pro-inflammatory mediators, such as prostaglandins (PGE_2_). These factors stimulate bladder sensory nerves and enhance detrusor muscle contraction, leading to postoperative pain, urgency or bladder spasm (18). Due to the acute onset and unpredictable intermittent nature of bladder spasms after TURBT, no clinical interventions could reliably reduce or completely prevent its occurrence. At present, its management focuses primarily on symptomatic relief (2). However, given the potential for serious consequences and the risk factors associated with bladder cancer itself, current therapeutic strategies require further optimization.

In the present study, our results demonstrated that for thulium laser ERBT, the incidence of postoperative CRBD (mild, moderate, or severe) was significantly lower as compared with conventional TURBT at 1 and 6h (*P* < 0.001), and the severity of CRBD was also significantly lower than in TURBT group at 1 and 6h (*P* < 0.001). The marked reduction in CRBD with thulium ERBT were considered to arise from its dual advantages: *en bloc* resection technique and minimized thermal injury.

During conventional TURBT, the tumor is cut in an “incise and scatter” manner, necessitates repeated resectoscope insertion and tumor fragmentation. Thus negative consequences might develop, such as risk of inadequate tumor clearance, mechanically irritating the trigone and bladder neck-regions densely innervated by M3 receptors. Furthermore, the electrocautery loop used in TURBT often causes tissue tearing and irregular wound surfaces, and the rough post-operative mucosa increases friction between the catheter and bladder wall. Conversely, during thulium ERBT, the tumor is removed in its entirety, encompassing the base, the underneath DM and a safety margin (“a no-touch technique”), thereby conforming to the oncological criteria of “optimized resection with low residual tumor rates” for cancer treatment. The performance of controlled cutting depth of thulium ERBT eliminates repetitive trauma, and its shallow tissue-penetration capacity creates smoother and more regular wound surfaces, hence preserving submucosal nerve plexuses and microvasculature. Histological studies have confirmed that ERBT leaves a 0.3–0.8 mm coagulation layer (while that datum is 2–3 mm for TURBT), reducing PGE2 and interleukin-6 (IL-6) release, both of which are the key mediators of bladder hypersensitivity (19). Moreover, the smoother bladder mucosa reduces physical irritation from the catheter, minimizing detrusor muscle overactivity and CRBD symptoms (20). This aligns with our finding of lower postoperative VAS scores in ERBT patients (1h: 2.39 ± 1.02 vs. 3.05 ± 1.25, *P* = 0.001; 6h: 2.03 ± 1.05 vs. 2.52 ± 1.26, *P* = 0.02).

During TURBT, broader tissue necrosis and subsequent encrustation are induced easier when using electrocautery. However, during laser ERBT, a “coagulation layer” is formed. Its wavelength is much closer to the water absorption peak value, so the absorbed energy rapidly coagulates small blood vessels (<1 mm in diameter) and lymphatic vessels during tissue cutting (21). The instantly coagulated tissue layer results in excellent hemostasis, spares surrounding normal mucosa and muscle layers, and reduces damage to sensory nerve endings (15). Thus, preservation of bladder wall structure and mucosal function decreases direct catheter-induced nerve stimulation. In our study, the shortened irrigation duration (ERBT group vs. TURBT group: 9.9 ± 1.9h vs. 11.1 ± 2.5h, *P* = 0.001) and fewer hematuria in the ERBT group were believed to stem from the thulium laser’s shallow tissue penetration and rapid vaporization-coagulation effects, minimizing thermal damage to surrounding vasculature.

Thermal damage remains a critical concern in transurethral surgical procedures, as excessive heat diffusion may lead to unintended tissue necrosis and postoperative complications, and prolonged recovery (22). Clinical studies have shown that thulium ERBT exhibited a significantly narrower thermal injury zone and minimized thermal damage (23). This advantage results from the unique biophysical properties of thulium laser. The thulium laser is strongly absorbed by water due to its specific wavelength, and the combination process of thulium laser with water can produce efficient thermal effect for tissue cutting, vaporization and coagulation during surgery, rendering precise incision. This contrasts sharply with the deeper thermal injury (one to three mm) caused by monopolar or bipolar electrocautery in TURBT, which can subsequently exacerbate bladder hypersensitivity and hypercontractility (21). Moreover, the shallow penetration depth of thulium laser reduces carbonization and necrosis at the resection site, as well as contributing to faster mucosal healing. Studies have demonstrated that reduced carbonization preserves the integrity of bladder mucosal layers (15), and its precision capacity ensures minimal disruption to the detrusor muscle and submucosal nerve plexuses. These collectively mitigate postoperative bladder wall irritation-key contributors to CRBD pathogenesis. In addition, laser ERBT does not generate any current flow, avoiding the occurrence of obturator nerve reflex, which is a greater advantage for BC patients in whom side wall tumors account for a large part (24). While for TURBT, stimulation of the obturator nerve reflex is a frequent side effect, causing leg jerking during surgery, which might result in iatrogenic bladder perforation (25), just as shown in our study.

Its better effect on CRBD may also be related to the reduced inflammatory response of thulium laser ERBT. During conventional TURBT, high-frequency currents generate extreme heat (300 °C–400 °C), causing deep tissue coagulation necrosis along with extensive thermal spread. Thus a local inflammatory response is triggered, resulting in edema of the bladder wall and subsequent infection, as well as release of pain-inducing factors that sensitize bladder afferent nerves (8). Contrarily, during thulium laser ERBT, a thin layer of tissue coagulation is formed and the degree of tissue inflammation or swelling is much lower. This difference has been verified by several studies that compared the inflammatory responses between laser ERBT and conventional TURBT (15). Jin Yongsheng et al. in a clinical study investigated the effects of laser ERBT on inflammatory factors (TNF-α, IL-6) in NMIBC patients in comparison with TURBT. Postoperatively, they found that the levels of TNF-α and IL-6 in ERBT group were lower than TRUBT group. They concluded laser ERBT had better efficacy than TURBT by reducing the inflammatory response of patients (26). Consequently, the minimal edema and inflammatory cell infiltration produced by laser ERBT not only minimize perioperative tissue necrosis, but also decrease bladder mucosal sensitivity, thus resulting in milder CRBD symptoms.

This study has several limitations that should be acknowledged. Firstly, its retrospective and single-center design introduces inherent potential for selection bias, as the choice of surgical procedure might be influenced by surgeon preference or patient-specific factors not fully accounted for. Secondly, the sample size, though sufficient for initial comparisons, was relatively modest, which might limit the generalizability of our findings and its power to detect differences in secondary outcomes or rare complications. Thirdly, the assessment of CRBD and pain, while using standardized scales (VAS, CRBD severity), relied on patient self-reporting and clinical evaluation, which could be subjected to interpretation bias, especially in elderly patients who might have difficulties distinguishing CRBD from surgical pain. Future studies could benefit from more objective biomarkers or urodynamic parameters. Fourthly, the standard TURBT in this study was performed using bipolar electrocautery. While this represents common clinical practice, the outcomes might differ if compared to other energy sources (such as holmium, green laser). Finally, the technical learning curve and resource requirements for thulium laser ERBT may pose challenges for widespread adoption, particularly in settings where TURBT is the established standard. Therefore, our results should be interpreted as promising but preliminary evidence, highlighting the need for rigorous, prospective, multicenter randomized controlled trials, as well as for evaluating its long-term oncological efficacy and cost-effectiveness.

## 5 Conclusion

Our retrospective analysis suggests that compared to conventional TURBT, thulium laser ERBT is associated with significant reductions in CRBD incidence and severity in the early postoperative period, lower postoperative pain scores, and improved patient satisfaction. The *en bloc* technique and the minimal thermal injury properties of the thulium laser are likely contributors to these benefits. However, given the retrospective nature and single-center setting of this study, more robust studies with larger cohorts and prospective, randomized designs are required to further validate these findings and to assess the long-term clinical impact of this technique.

## Data Availability

The raw data supporting the conclusions of this article will be made available by the authors, without undue reservation.

## References

[1] Jang EB Hong SH Kim KS Park SY Kim YT Yoon YE Catheter-related bladder discomfort: how can we manage it? Int Neurourol J (2020) 24(4):324–31. 10.5213/inj.2040108.054 33401353 PMC7788325

[2] Markopoulos T Katsimperis S Lazarou L Tzelves L Mitsogiannis I Papatsoris A Catheter-related bladder discomfort: insights into pathophysiology, clinical impact, and management. Cureus (2025) 17:e81322. 10.7759/cureus.81322 40291191 PMC12034329

[3] Park JY Hong JH Yu J Kim DH Koh GH Lee SA Effect of ketorolac on the prevention of postoperative catheter-related bladder discomfort in patients undergoing robot-assisted laparoscopic radical prostatectomy: a randomized, Double-Blinded, placebo-controlled Study. J Clin Med (2019) 8(6):759. 10.3390/jcm8060759 31146434 PMC6616938

[4] Zhou L Zhou L Tian L Zhu D Chen Z Zheng C Preoperative education with image illustrations enhances the effect of tetracaine mucilage in alleviating postoperative catheter-related bladder discomfort: a prospective, randomized, controlled study. BMC Anesthesiol (2018) 18(1):204. 10.1186/s12871-018-0653-y 30579342 PMC6303915

[5] Mitobe Y Yoshioka T Baba Y Yamaguchi Y Nakagawa K Itou T Predictors of catheter-related bladder discomfort after surgery: a literature review. J Clin Med Res (2023) 15(4):208–15. 10.14740/jocmr4873 37187710 PMC10181350

[6] Hou J Li Y Wu Y Liu Y Chen Q Li Y Safety and efficacy of wrist-ankle acupuncture in treating catheter-related bladder discomfort after transurethral resection of the prostate: a double-blind randomized clinical trial. Gland Surg (2022) 11(9):1464–71. 10.21037/gs-22-438 36221271 PMC9547715

[7] Huh H Lee SW Cho JE Kim HC . Effect of chlorpheniramine administration on postoperative catheter-related bladder discomfort in patients undergoing transurethral excision of bladder tumor: a prospective randomized study. J Anesth (2021) 35(5):646–53. 10.1007/s00540-021-02970-4 34245368

[8] Ergenoglu P Akin S Yalcin Cok O Eker E Kuzgunbay B Turunc T Effect of intraoperative paracetamol on catheter-related bladder discomfort: a prospective, randomized, double-blind Study. Curr Ther Res (2012) 73(6):186–94. 10.1016/j.curtheres.2012.08.001 24653520 PMC3955106

[9] Fan B Shen J Wu L Zhang P . Study of mirabegron and solifenacin in the improvement of catheter-related bladder discomfort in patients undergoing transurethral resection: a case–control study. Medicine (Baltimore) (2022) 101(48):e32052. 10.1097/md.0000000000032052 36482620 PMC9726409

[10] Jendoubi A Aissi W Abbes A Bouzouita A Fourati S Necib H Efficacy and safety of Parecoxib for prevention of catheter-related bladder discomfort in patients undergoing transurethral resection of bladder tumor: prospective randomised trial. Indian J Anaesth (2018) 62(6):461. 10.4103/ija.ija_137_18 29962529 PMC6004747

[11] Zugail AS Pinar U Irani J . Evaluation of pain and catheter-related bladder discomfort relative to balloon volumes of indwelling urinary catheters: a prospective study. Investig Clin Urol (2019) 60(1):35. 10.4111/icu.2019.60.1.35 30637359 PMC6318203

[12] Lin CH Lu IC Gau TP Cheng KI Chen HL Hu PY . Preventing postoperative catheter-related bladder discomfort (CRBD) with bladder irrigation using 0.05% lidocaine saline solution: monitoring with Analgesia Nociception Index (ANI) after transurethral surgery. Medicina (Mex). (2024) 60(9):1405. 10.3390/medicina60091405 39336446 PMC11433757

[13] Mahesh C. T Pirzada F. M Rajeev S Anurag S Nikhil K Rajpal S A prospective study comparing side-firing KTP laser enucleation vs. bipolar transurethral resection of bladder tumor for small bladder tumors in an outpatient setting. Cent Eur J Urol (2021) 74(2). 10.5173/ceju.2021.0012.R1 PMC8318012 34336241

[14] Badawy A Sultan SM Marzouk A El-Sherif E . Thulium laser *en bloc* resection *versus* conventional transurethral resection of urinary bladder tumor: a comparative prospective study. Urol Ann (2023) 15(1):88–94. 10.4103/ua.ua_59_22 37006212 PMC10062500

[15] Gao W . Current laser application in *En bloc* resection of bladder tumor-a narrative literature review. World J Surg Oncol (2025) 23(1):165–75. 10.1186/s12957-025-03815-0 40287765 PMC12034164

[16] Gao W . Current opinions regarding the clinical utility of *en bloc* resection in the treatment of non-muscle invasive bladder cancer—a review of the literature. Discover Oncol (2024) 15(1):574. 10.1007/s12672-024-01452-9 39425810 PMC11490474

[17] Park JY Baek JW Yu J Kim CS Bae J Kim YK . Vitamin C and catheter-related bladder discomfort after transurethral resection of bladder tumor: a double-blind, randomized, placebo-controlled study. J Clin Anesth (2023) 89:111191. 10.1016/j.jclinane.2023.111191 37356194

[18] Shim JW Cha S Moon HW Moon YE . Effects of intraoperative magnesium and Ketorolac on catheter-related bladder discomfort after transurethral bladder tumor resection: a prospective randomized Study. J Clin Med (2022) 11(21):6359. 10.3390/jcm11216359 36362587 PMC9659173

[19] Wang X Ni X Zhao S Zhao R Wang X Xia S ROS–NLRP3 signaling pathway induces sterile inflammation after thulium laser resection of the prostate. J Cell Physiol (2022) 237(3):1923–35. 10.1002/jcp.30663 35023144

[20] Yang H Wang N Han S Male M Zhao C Yao D Comparison of the efficacy and feasibility of laser enucleation of bladder tumor *versus* transurethral resection of bladder tumor: a meta-analysis. Lasers Med Sci (2017) 32(9):2005–12. 10.1007/s10103-017-2308-5 28831631

[21] Liu Z Zhang Y Sun G Ouyang W Wang S Xu H Comparison of thulium laser resection of bladder tumors and conventional transurethral resection of bladder tumors for non-muscle-invasive bladder cancer. Urol Int (2022) 106(2):116–21. 10.1159/000514042 33784709

[22] Croghan SM Compton N Manecksha RP Cullen IM Daly PJ . *En bloc* transurethral resection of bladder tumors: a review of current techniques. Can Urol Assoc J (2022) 16(5):E287–E293. 10.5489/cuaj.7539 34941487 PMC9119597

[23] Assem A Kassem A Sherif M Lotfi A Abdelwahed M . Safety, feasibility, and quality of thulium laser *en-bloc* resection for treatment of non-muscle invasive bladder cancer. Int Urol Nephrol (2023) 55(12):3103–9. 10.1007/s11255-023-03752-5 37639155 PMC10611837

[24] Petov V Timofeeva E Sukhanov R Baniel J Mustafin M Fajkovic H Prospective non-randomized comparison of transurethral laser *en bloc* resection vs. conventional resection of bladder tumors larger than 3 cm. Minerva Urol Nephrol (2024) 76(4):436–41. 10.23736/s2724-6051.24.05682-9 39051891

[25] Zhang W Zhou B Deng J Han G Ni W Nie Q . Retrospective analysis of 1470-/980-nm dual-wavelength laser *en bloc* resection *versus* transurethral resection of bladder tumor for primary non-muscle-invasive bladder cancer. Lasers Med Sci (2023) 38(1):44. 10.1007/s10103-023-03708-2 36656398

[26] Jin Y Liangliang H Jiawei Z Gang W . Effects of transurethral holmium laser resection on miR-119a, miR-200b and inflammatory factors in patients with high-risk non-muscular invasive bladder cancer. Int J Urol Nephrol (2024)(5) 451–5.

